# Is the ghost of brown tumor back again? Features of hypercalcaemic primary hyperparathyroidism we must not forget

**DOI:** 10.1007/s12020-021-02896-7

**Published:** 2021-10-09

**Authors:** Irene Gagliardi, Alessandro Mella, Paola Franceschetti, Maria Rosaria Ambrosio, Maria Chiara Zatelli

**Affiliations:** 1 Section of Endocrinology, Geriatrics and Internal Medicine, Department of Medical Sciences, University of Ferrara, Ferrara, Italy; 2 Unit of Endocrinology and Metabolic Diseases – Oncology and Specialty Medicines Department, Azienda Ospedaliero Universitaria di Ferrara, Ferrara, Italy

Two patients presenting with mandible-jaw neoformations were referred to us for endocrine evaluation. A 51-year-old male presented with painful T9 vertebral fracture and multiple osteolytic areas in spine, pelvis, and right mandible branch (Fig. 1A). Metastatic disease was excluded with ^18^fluorine-fluorodeoxyglucose positron emission tomography (^18^F-FDG-PET) showing multiple uptakes corresponding to osteolytic areas. Mandible lesion biopsy was not conclusive. Biochemical workup was consistent with hypercalcaemic primary hyperparathyroidism (HPH) (calcium 13.4 mg/dl, range: 8.5–10.5 mg/dl; PTH 667 pg/ml, range 12–88 pg/ml) and vitamin D deficiency (7.8 ng/ml). Neck ultrasound (US) performed at our Unit showed a hypoechoic area under the left thyroid lobe suspected for enlarged parathyroid. This lesion was also identified after CT images re-evaluation (Fig. 1B). Pathology review of the previously provided sample integrated with clinical and radiological information was finally concordant with brown tumor (BT) of the jaw. Spine magnetic resonance was performed and confirmed multiple osteolytic spine lesions typical of BT (Fig. 1C). Left inferior parathyroidectomy was performed and histology showed a parathyroid adenoma. After surgery, serum calcium levels rapidly dropped. High-dose oral and parenteral calcium supplementation as well as vitamin D active metabolite administration was needed in order to contrast hungry bone syndrome development. Concerning vertebral fractures, a corset was applied for at least 1 month and therapy with cholecalciferol was subsequently started. In addition, further treatment with zoledronic acid (5 mg) was administered in order to prevent further fractures.

**Fig. 1:**
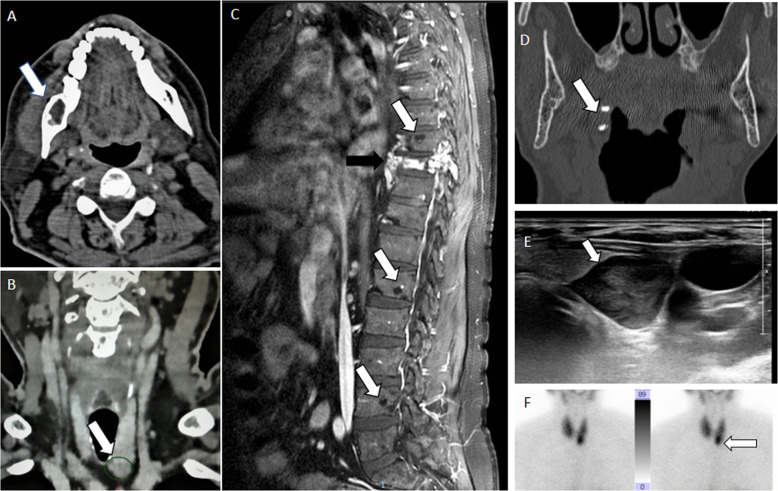
Brown tumor and parathyroid radiological workup. Patient 1**:** **A** CT image of the 2 cm mandibular lesion (white arrow); **B** CT image of the enlarged parathyroid (white arrow); **C** MRI image of vertebral BT (white arrows) and D9 vertebral fracture (black arrow). Patient 2: **D** dental CT images showing right pharyngeal wall calcifications (white arrow); **E** enlarged parathyroid at neck US (white arrow); **F** parathyroid Technetium (^99^mTc) sestamibi scintigraphy showing hyperfunctioning parathyroid gland under the left thyroid lobe (white arrow)

The second case was a 68-year-old woman complaining of mildly painful swelling in the left maxillary branch. Dental CT showed a hypodense lesion at 25, 26, and 27 alveolar regions and small collateral calcifications in the right pharyngeal wall (Fig. 1D). Lesion biopsy was concordant with a giant cell granuloma. Afterwards, biochemical workup showed severe HPH (calcium 15 mg/dl; PTH 898 pg/ml) and vitamin D deficiency (9.8 mg/dl). Neck US performed at our Unit highlighted a hypoechoic and hypervascularized nodule highly suspected for an enlarged left parathyroid (Fig. 1E), then confirmed by scintigraphy (Fig. 1F). Zoledronic acid was started at 4 mg iv, followed by left inferior parathyroidectomy. Histology confirmed a parathyroid adenoma. After surgery, normal calcium levels were obtained by only oral calcium calcitriol and cholecalciferol supplementation. Maxillary swelling progressively decreased, suggesting that the initial histological diagnosis of giant cell granuloma was wrong.

Available HPH guidelines do not provide clear indications concerning BT presentation and management [1]. BT appears as a uni- or multi-lobular osteolytic area with soap-bubbling features on X-rays. On CT images, BT looks as a well-defined osteolytic area, with possible fluid signal, rarely showing soft tissue invasion. BT radiological differential diagnosis could be difficult, especially with bone metastases [2]. BT histological diagnosis needs to be integrated with radiological, biochemical, and clinical findings to get the correct diagnosis and avoid expensive second-level imaging, such as ^18^F-FDG-PET. These two clinical cases underline the importance of evaluating calcium homeostasis in patients presenting with osteolytic facial bone mass suspected for BT. Once HPH is biochemically detected, US is very useful to identify neck masses consistent with enlarged parathyroids that could be confirmed by subsequent scintigraphy [3]. Literature indicates that BT management is mainly conservative since BT usually regresses after hyperparathyroidism treatment. However, growing BT may require surgical management [2].

In conclusion, our cases underline the importance of suspecting HPH in patients with low-to-mild aching bone masses in skeletal regions with major cortical components clinically and radiologically consistent with BT. Patients should undergo imaging workup as well as clinical and biochemical investigation. Multidisciplinary management is advisable.

## Data Availability

The data can be made available by the corresponding author upon request.
